# Clinical evaluation of AI-assisted muscle ultrasound for monitoring muscle wasting in ICU patients

**DOI:** 10.1038/s41598-024-64564-w

**Published:** 2024-06-26

**Authors:** Phung Tran Huy Nhat, Nguyen Van Hao, Lam Minh Yen, Nguyen Hoang Anh, Dong Phu Khiem, Hamideh Kerdegari, Le Thanh Phuong, Vo Tan Hoang, Nguyen Thanh Ngoc, Le Ngoc Minh Thu, Truong Ngoc Trung, Luigi Pisani, VITAL Consortium, Liane Canas, Alberto Gomez, Hamideh Kerdegari, Andrew King, Marc Modat, Reza Razavi, Miguel Xochicale, Dang Phuong Thao, Dang Trung Kien, Doan Bui Xuan Thy, Dong Huu Khanh Trinh, Du Hong Duc, Ronald Geskus, Ho Bich Hai, Ho Quang Chanh, Ho Van Hien, Huynh Trung Trieu, Evelyne Kestelyn, Le Dinh Van Khoa, Le Thuy Thuy Khanh, Luu Hoai Bao Tran, Luu Phuoc An, Angela Mcbride, Nguyen Lam Vuong, Nguyen Quang Huy, Nguyen Than Ha Quyen, Nguyen Thi Giang, Nguyen Thi Diem Trinh, Nguyen Thi Le Thanh, Nguyen Thi Phuong Dung, Nguyen Thi Phuong Thao, Ninh Thi Thanh Van, Pham Tieu Kieu, Phan Nguyen Quoc Khanh, Phung Khanh Lam, Guy Thwaites, Louise Thwaites, Tran Minh Duc, Trinh Manh Hung, Hugo Turner, Jennifer Ilo Van Nuil, Vu Ngo Thanh Huyen, Sophie Yacoub, Cao Thi Tam, Duong Bich Thuy, Ha Thi Hai Duong, Ho Dang Trung Nghia, Le Buu Chau, Le Mau Toan, Le Ngoc Minh Thu, Le Thi Mai Thao, Luong Thi Hue Tai, Nguyen Hoan Phu, Nguyen Quoc Viet, Nguyen Thanh Dung, Nguyen Thanh Nguyen, Nguyen Thanh Phong, Nguyen Thi Kim Anh, Nguyen Van Hao, Nguyen Van Thanh Duoc, Pham Kieu Nguyet Oanh, Phan Thi Hong Van, Phan Tu Qui, Phan Vinh Tho, Truong Thi Phuong Thao, Luigi Pisani, Marcus Schultz, Natasha Ali, David Clifton, Mike English, Jannis Hagenah, Ping Lu, Jacob McKnight, Chris Paton, Tingting Zhu, Linda Denehy, Thomas Rollinson, Pantelis Georgiou, Bernard Hernandez Perez, Kerri Hill-Cawthorne, Alison Holmes, Stefan Karolcik, Damien Ming, Nicolas Moser, Jesus Rodriguez Manzano, Walter Karlen, Reza Razavi, Sophie Yacoub, Nguyen Van Vinh Chau, Andrew P. King, Louise Thwaites, Linda Denehy, Alberto Gomez

**Affiliations:** 1 School of Biomedical Engineering Imaging Sciences, King’s College London, London, UK; 2 Oxford University Clinical Research Unit, Ho Chi Minh City, Vietnam; 3 Hospital for Tropical Diseases, Ho Chi Minh City, Vietnam; 4 National Hospital for Tropical Diseases, Hanoi, Vietnam; 5 Mahidol Oxford Research Unit, Bangkok, Thailand; 6 Centre for Tropical Medicine and Global Health, University of Oxford, Oxford, UK; 7 University of Melbourne, Melbourne, Australia; 8 Imperial College London, London, UK; 9 University of Ulm, Baden-Württemberg, Germany

**Keywords:** Muscle ultrasound, Muscle wasting, Intensive care unit, Artificial intelligence, Real-time, Biomedical engineering, Muscle, Ultrasonography, Translational research

## Abstract

Muscle ultrasound has been shown to be a valid and safe imaging modality to assess muscle wasting in critically ill patients in the intensive care unit (ICU). This typically involves manual delineation to measure the rectus femoris cross-sectional area (RFCSA), which is a subjective, time-consuming, and laborious task that requires significant expertise. We aimed to develop and evaluate an AI tool that performs automated recognition and measurement of RFCSA to support non-expert operators in measurement of the RFCSA using muscle ultrasound. Twenty patients were recruited between Feb 2023 and July 2023 and were randomized sequentially to operators using AI (n = 10) or non-AI (n = 10). Muscle loss during ICU stay was similar for both methods: 26 ± 15% for AI and 23 ± 11% for the non-AI, respectively (*p* = 0.13). In total 59 ultrasound examinations were carried out (30 without AI and 29 with AI). When assisted by our AI tool, the operators showed less variability between measurements with higher intraclass correlation coefficients (ICCs 0.999 95% CI 0.998–0.999 vs. 0.982 95% CI 0.962–0.993) and lower Bland Altman limits of agreement (± 1.9% vs. ± 6.6%) compared to not using the AI tool. The time spent on scans reduced significantly from a median of 19.6 min (IQR 16.9–21.7) to 9.4 min (IQR 7.2–11.7) compared to when using the AI tool (*p* < 0.001). AI-assisted muscle ultrasound removes the need for manual tracing, increases reproducibility and saves time. This system may aid monitoring muscle size in ICU patients assisting rehabilitation programmes.

## Introduction

There is a large body of research demonstrating that patients admitted to the intensive care unit (ICU) suffer significant morbidity including functional impairments and early and rapid loss of muscle mass^1^. Loss of muscle mass contributes to muscle dysfunction and may impact overall function, but other variables are also implicated. The reasons for these changes are multifactorial and may include impaired muscle protein synthesis associated with sepsis^2^; patient comorbidity, organ dysfunction, duration of mechanical ventilation and length of ICU stay.

Measurement of muscle changes in ICU is challenging due to patient sedation and subsequent difficulties with following commands when using traditional volitional techniques such as the Medical Research Council sum score^3^. The use of a non-volitional measure such as point-of-care ultrasound (POCUS) offers the potential to examine muscle changes^4^.

Patients admitted with tetanus, of whom 50% are intubated and ventilated, administered muscle relaxant drugs and benzodiazepines, spend approximately three weeks in the ICU^5^. Patient index admission diagnosis and sequelae from the ICU admission (such as sepsis) are associated with loss of muscle mass, weakness and impaired functional outcomes^6^. Assessment of function is difficult in these patients. The use of POCUS has gained traction and in patients with muscle failure such as tetanus allows serial monitoring.

Muscle ultrasound is commonly used to monitor muscle wasting by measuring the Rectus Femoris cross-sectional area (RFCSA)^6,7^. In a recent systematic review, muscle ultrasound was used in 85% (28/33) of the studies to assess muscle mass in the ICU^8^. The effectiveness of ultrasound for assessing muscle mass is comparable to, but safer, cheaper, more accessible and more easily repeatable than any other imaging modality in the ICU^9–12^. In this study, we focused on the RFCSA for several reasons. Firstly, the RFCSA is easily identifiable through its size and location, providing a reliable indicator of muscle mass and strength. Studies, including research by Puthucheary et al.^11^, have demonstrated that measurements of muscle thickness can significantly underestimate muscle wasting in ICU patients compared to RFCSA assessments. Moreover, RFCSA has been shown to correlate more closely with muscle strength, which is particularly relevant in the ICU where patient cooperation may be limited. Given these advantages, we opted to utilize an AI-guided tool to measure RFCSA. In addition, recent evidence suggests that the assessment of changes in muscle size over time may improve prognostication and enhance the choice of rehabilitation interventions that may address muscle wasting^4,11,13^. However, the process of measurement of RFCSA from ultrasound is a time-consuming task and often suffers from significant intra and interobserver variability^10,11,14^, hindering its use to inform patient management. When applied to the exact same stand views, repeated RFCSA muscle recordings always introduce image differences due to variations in probe positioning, angulation, and tilt, as well as the manual delineation of muscle by the operator. Conventionally, to enhance the accuracy of the measurement of the muscle size, the RFCSA is measured three times consecutively and an average of the measurements is calculated and saved, which increases the time taken for scanning and data acquisition.

To address the challenge of intra- and interobserver variability in muscle ultrasound measurement, AI techniques have been proposed^15–17^. However, the main limitation with these tools is that they have typically been designed and evaluated for offline use, i.e., they are not suitable for bedside use and real-time analysis. Moreover, to date these tools have been subject to limited validation in clinical settings.

**Figure 1:**
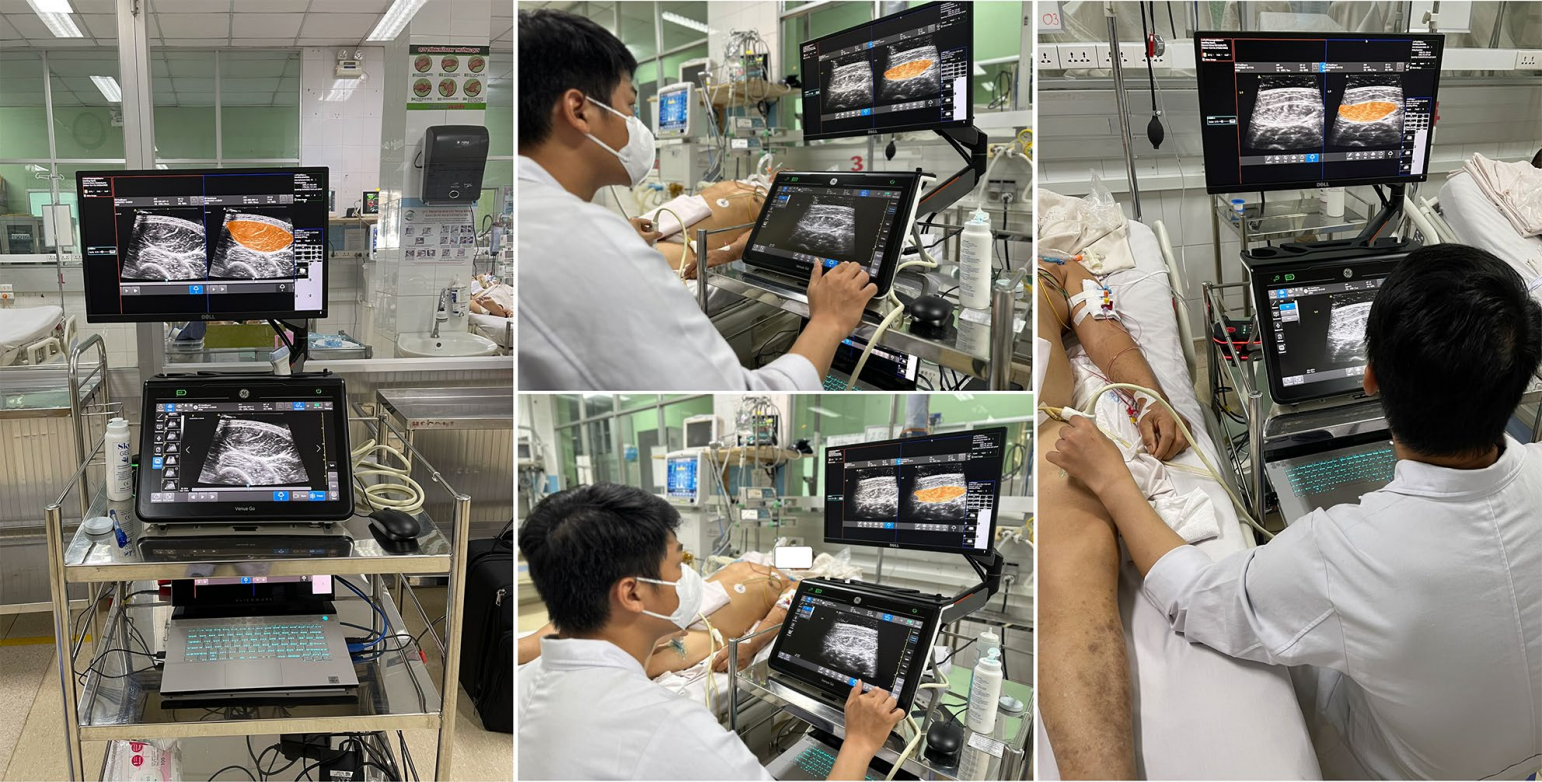
Real-time AI-assisted muscle ultrasound (RAIMUS) system.

In this study, we aimed to investigate the feasibility of using a real-time AI-assisted tool for RFCSA measurement from muscle ultrasound that would be suitable for clinical use, particularly in an LMIC setting. We hypothesized that this tool would have improved reproducibility and reduced interobserver variability compared to current methods. We tested our tools in a cohort of patients with tetanus as this is a group of patients in whom muscle wasting is an important problem with long ICU stays and in whom such a tool would be used if proved reliable (Fig. 1).

## Methods

This was a prospective observational study to test the reliability of AI-assisted measurement of RFCSA from muscle ultrasound at the patient's bedside compared to standard ultrasound. The study was conducted in the adult ICU at the Hospital for Tropical Diseases Ho Chi Minh City, Vietnam. Measurements were performed in adult patients with severe tetanus (Ablett Grade 3 or 4) admitted to the Adult ICU at HTD expected to stay at least 5 days. Patients were receiving standard treatment including mechanical ventilation, muscle relaxation and neuromuscular blockers following the hospital guideline^5^. All patients or their representatives provided informed consent to take part in the study. Ethics approval was obtained from the HTD Ethics Committee, and the Oxford Tropical Research Ethics Committee (OxTREC). The work was adherent to the tenets of the Declaration of Helsinki. The study was registered at ClinicalTrials.gov number NCT06034093 on 13/09/2023.

**Figure 2:**
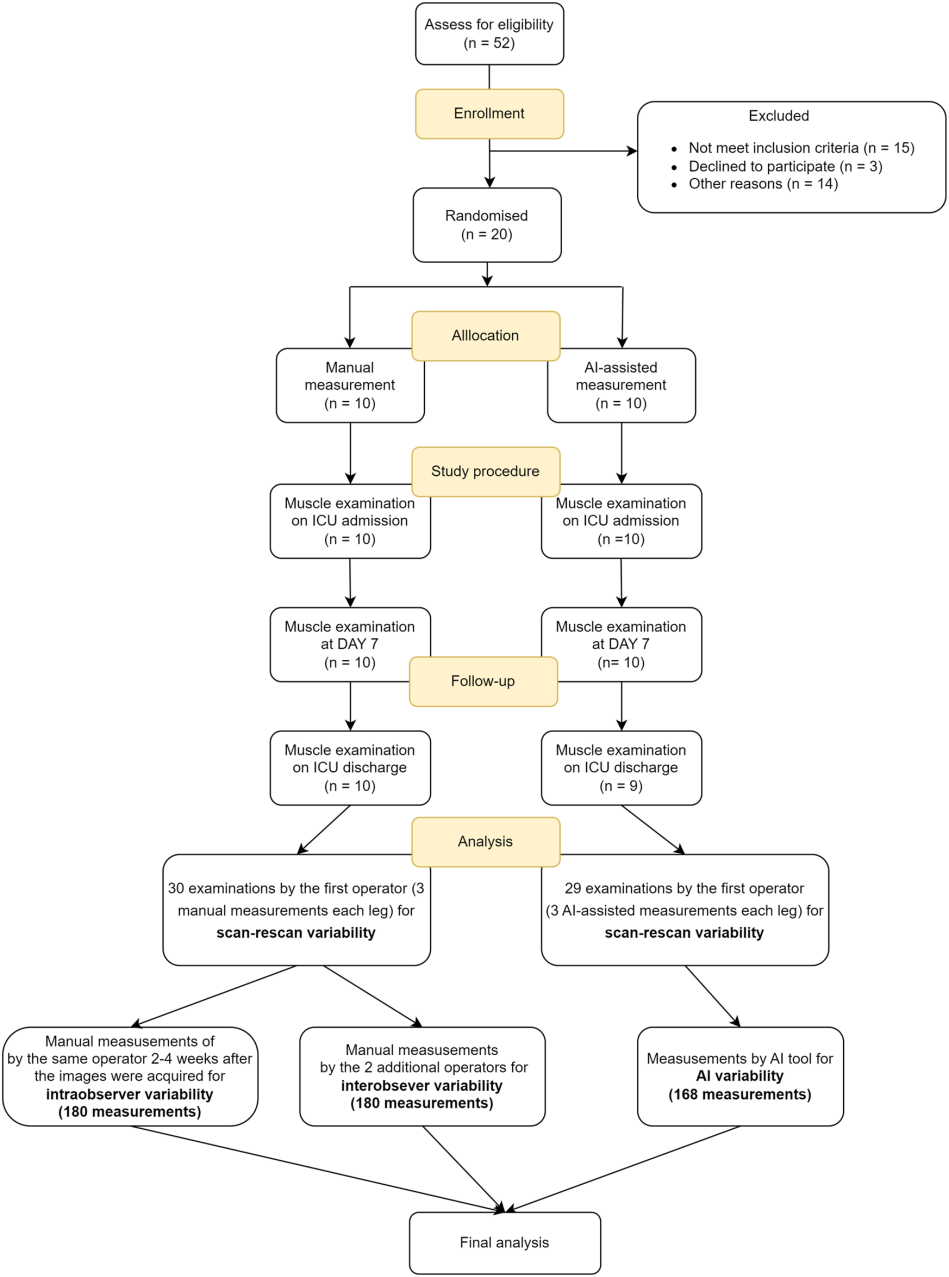
Study flowchart.

RFCSA measurements were by using a standard technique or the AI-assisted method based on patient allocations—where patients were randomly assigned to receive either standard RFCSA measurements or AI-enabled measurements at regular intervals during their ICU stay (see Fig. 2).

All measurements were carried out according to a standard operating procedure where patients were in the supine position with the leg in neutral rotation^6,18^. Patients not receiving muscle relaxants were reminded to relax the muscle. Measurements were taken using a 12L-RS linear probe and a Venue Go ultrasound machine (General Electric Healthcare, London, UK). Measurements were taken from a location which was three-fifths of the distance from the anterior superior iliac spine to the superior patella pole. This position was used as a landmark for subsequent measurements to provide consistency and allow reliable comparisons to be made over time. The transducer was placed perpendicular to the skin and transversally in relation to the longitudinal axis of the thigh to observe the cross-sectional area of the muscle. An excess of ultrasound gel was placed when performing the muscle scan and the pressure on the skin was kept minimal to ensure good image quality^6,8,18^. For each examination, 3 separate measurements (scan-rescan) were made for each leg (removing the probe between each one). The examinations’ durations (including all 3 scans) were recorded.

The operators selected were 3 clinicians and 2 nurses, all 5 with limited training in muscle ultrasound as our target users for the AI tool are non-expert operators to reflect the common setting in a LMIC. We provided muscle ultrasound training and RAIMUS software training to allow operators to use the tool effectively. Each operator was asked to scan five patients (2 legs, each leg 3 repeat scans) as part of their muscle training with the AI software. The images were saved and manually delineated then automatically measured with the AI software.

For patients in the standard measurement arm, RFCSA was determined by manual delineation of the cross-sectional image of the muscle. For patients in the AI-assisted imaging arm measurements were made in real time using the automated AI tool.

The AI-enabled technique using RAIMUS software is described in detail in the Supplementary File 1 and illustrated in Fig. 2. Our RAIMUS software enables automated detection and measurement of RFCSA which can be visualized in real time using the Plugin-based REal Time UltraSound software platform (PRETUS) which connects a laptop to the ultrasound machine duplicating the ultrasound image display and superimposing the AI delineation measurement tool^19^. Once the probe is in the right position and the clinician is satisfied with the quality of the image/AI-generated segmentation mask, they store the image as a standard view. Next, the RFCSA is delineated by the AI tool or by the operator using a movable cursor following the inner echogenic line of the rectus fascia.

### Evaluation of reproducibility

We assessed three types of variability including (1) scan-rescan variability, (2) intraobserver variability in delineation and (3) interobserver variability in delineation (Fig. 1). To assess (1) scan-rescan variability, the operators were asked to scan each leg three times for each of the two allocated methods. To assess (2) intraobserver variability in delineation over time, the same operator subsequently delineated each image one further time 2–4 weeks after the images were acquired (from stored raw images). To assess (3) interobserver variability in delineation, each image acquired by the first operator was delineated by 2 additional operators.

The examination durations (measured from when the operators put the probe on the leg of patient to when they finished delineations and measurements) were compared between the 2 methods. To evaluate usability, a questionnaire was administered to the operators at the end of the procedure (Supplementary File 1).

### Sample size

The sample size for this study was estimated following Walter et al.^20^ with the minimum acceptable reliability (Intraclass correlation coefficient ICC) (ρ_0_) of 0.9, expected reliability (ICC) (ρ_1_) of 0.96, significance level two-tailed (α) of 0.05, Power (1 − β) of 80% and the number of raters/measurements per subjects (k) of 3. After using the formula, the sample size was 27 examinations. With the expected dropout of 10% the total sample size used was 30 examinations for each group.

### Statistical analysis

All statistical analyses were performed with R version 4.0.4. Continuous variables are expressed as mean ± standard deviation (SD) or as median (interquartile range), according to the symmetricality of the data distribution, and compared using an unpaired Student’s t-test or Wilcoxon rank sum test, as appropriate. Categorical data, presented as numbers and percentages, were compared using the χ2 test. *P* values less than 0.05 were considered statistically significant.

The variability in RFCSA measurements were assessed using the two-way random effects for multiple raters/measurements ICC with 95% CI (ICC (2, k)^21^. The ICC is a measure of reliability, specifically the reliability of two different raters to measure subjects similarly, with numbers closest to 1 representing a high similarity of measurements between measurements. The standard error of measurement (SEM) was also calculated to make a judgment about the degree that measurements vary for an individual. The SEM values indicate the precision of the measurement and were calculated based on the ICC and the SD of the mean of differences between the two measurements SEM = SD√1—ICC. There was no measure-remeasure variation for the automated AI software because the model always outputs the same measurement and hence the same RFCSA result. A modified Bland–Altman analysis for multiple observers in a single plot proposed by^22^ was used to assess the agreement between RFCSA measurements. The examination duration was compared between the two methodologies of measurement using an unpaired Student’s t-test.

### Ethics declarations

This study was approved by the Oxford Tropical Research Ethics Committee (OxTREC) and the HTD Institutional Review Boards.

## Results

### Patients

We enrolled 20 patients with tetanus at the Adult ICU at HTD between Feb 2023 and July 2023. The mean ± SD age of patients in the AI group and non-AI group were 67 ± 13 years and 56 ± 17 years, respectively. Two (20%) patients in each group were female. 17 (85%) patients had at least one episode of hospital acquired infection (HAI) during ICU stay. The ICU stay and hospital stay were comparable between the two groups (Table 1).

**Table 1:** Characteristics of patients in the study (n = 20).

	AI arm (n = 10)	Non-AI arm (n = 10)
Age	67 ± 13	56 ± 17
Sex (female)	2 (20%)	2 (20%)
Weight (kg)	55.9 ± 9.6	52.4 ± 14.0
Height (cm)	163.2 ± 7.0	161.3 ± 6.8
BMI (kg/m^2^)	20.9 ± 2.4	20.0 ± 5.0
Comorbidities (1 or more)	6 (60%)	4 (40%)
Hypertension	6 (60%)	4 (40%)
Diabetes	1 (10%)	0 (0%)
Others (Chronic liver disease, arthritis)	1 (10%)	1 (10%)
Sedative use during ICU	10 (100%)	10 (100%)
Use of non-depoplarising neuromuscular blocking agents during ICU stay	10 (100%)	10 (100%)
Length of ICU stay (days)	26 ± 11	24 ± 5
Length of hospital stay (days)	32 ± 13	31 ± 6
Mechanical ventilation duration (days)	19.7 ± 8.2	17.8 ± 5.5
ANSD duration (days)	12.4 ± 6.7	12.0 ± 2.8
Total dose of Pipecuronium	438 ± 190	430 ± 250
Enteral nutrition	10 (100%)	10 (100%)
Rehabilitation duration (days)	10 ± 9	8 ± 4
HAI during ICU stay	8 (80%)	9 (90%)
RF CSA D1 (cm^2^)	4.37 ± 1.08	4.76 ± 1.50
RF CSA D7 (cm^2^)	4.09 ± 1.01	4.51 ± 1.59
RFCSA Discharge (cm^2^)	3.25 ± 1.24	3.65 ± 1.15
% change in RFCSA during ICU stay (%)	26 ± 15	23 ± 11
ICU survival	10 (100%)	10 (100%)

*BMI* body mass index, *MV* mechanical ventilation, *ANSD* autonomous nervous system dysfunction, *HAIs* hospital-acquired infections, *RFCSA D1* rectus femoris cross sectional area on ICU admission, *RFCSA D7* rectus femoris cross sectional area on day 7, *RFCSA Discharge* rectus femoris cross sectional area at ICU discharge.

### Ultrasound examinations and reproducibility

In total 59 muscle ultrasound examinations were carried out, 29 examinations with the AI tool and 30 examinations without the AI tool. After visual inspection of 29 examinations with AI, 28 examinations were successfully delineated by the AI tool and one examination was rejected by the expert. All examinations without the AI tool were accepted by the expert. The average muscle loss during ICU was similar in the two groups, 26 ± 15% for the AI arm and 23 ± 11% for the non-AI arm. The full characteristics of the patients are shown in Table 1.

#### Scan-rescan variability

The scan re-scan variability of the AI group was lower compared to the non-AI group (ICC 0.999 95%CI 0.998–0.999, vs ICC 0.982 95%CI 0.962–0.993). (Supplementary File Table S2).

**Figure 3:**
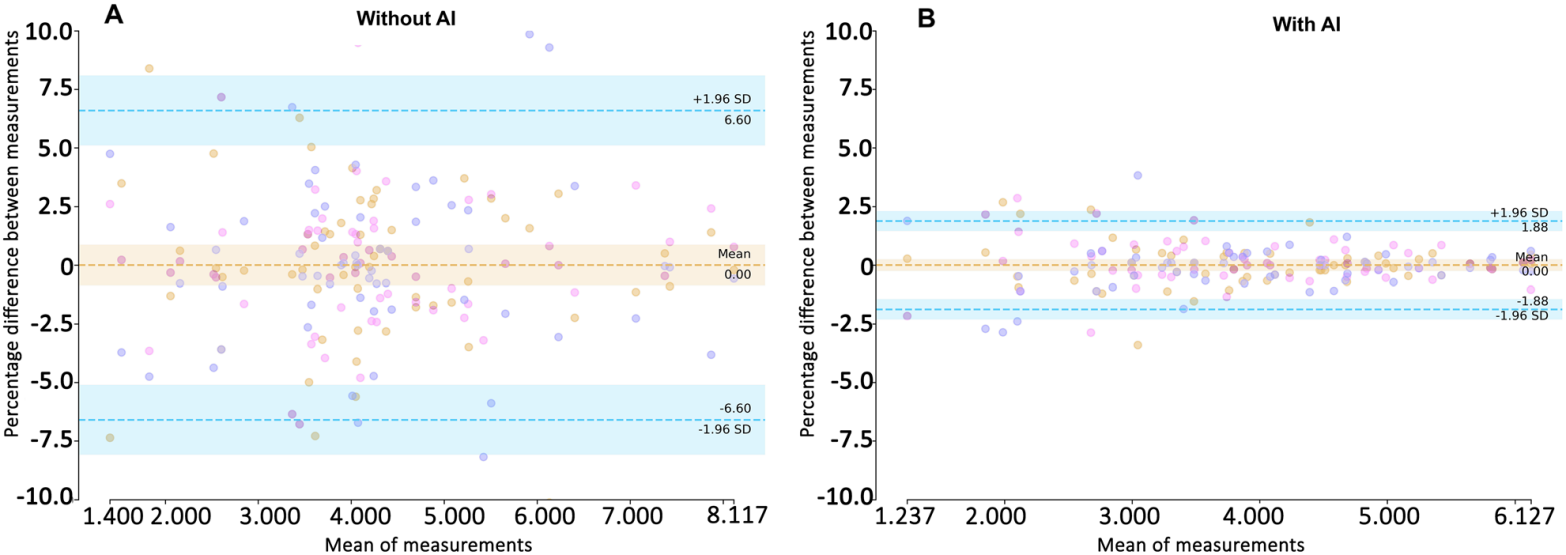
Plot of scan-rescan agreement in RFCSA. (**A**) Without AI. (**B**) With AI. Horizontal dotted lines indicate the limits of agreement from the mean (LoA) of the three measurements. Some symbols are superimposed. The percentage differences of all measurements with the mean (y-axis) are plotted against the mean RFCSA for all participants (x-axis). The horizontal dashed lines indicate the limits of agreement with the mean of the three repeated measurements and ranged from −6.6 to 6.6% for the non-AI group and −1.9 to 1.9% for the AI group.

Figure 3 shows modified Bland–Altman plots illustrating the percentage difference in three repeated RFCSA measurements from the mean. The figures showed better agreement in the AI arm with the limits of agreement were lower in the AI group (± 1.9%) compared to the non-AI group (± 6.6%).

#### Intraobserver variability in delineation over time

**Figure 4:**
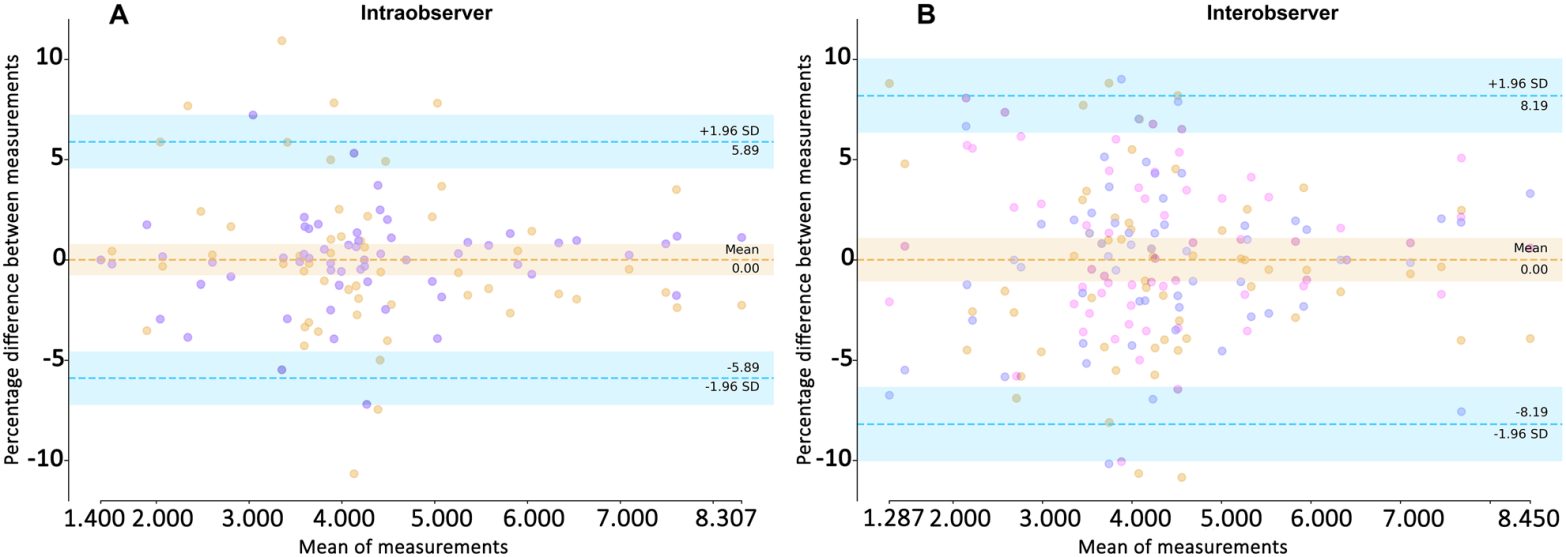
Intraobserver and Interobservers agreement plots of RFCSA measurements: (**A**) of the same operator over time and (**B**) interobserver agreement plot between 3 observers. Observers represent different symbols. The percentage differences of all measurements with the mean (y-axis) are plotted against the mean RFCSA for all participants (x-axis). The horizontal dashed lines indicate the limits of agreement with the mean of the three observers and ranged from −5.9 to 5.9% for interobserver (left) and from −8.2 to 8.2% for interobserver (right).

The manual intraobserver in delineation (initial vs 2–4 weeks later) resulted in good reliabity with an ICC of 0.984 (95% CI 0.973, 0.990). The modified Bland Altman plot for intraobserver agreement results is shown in Fig. 4 (left). The intraobserver agreement without AI was ± 5.9%.

#### Interobserver variability in delineation

The manual interobserver ICC was 0.974 (95% CI 0.965–0.981). The interobserver agreement results are shown in Fig. 4 (right). The limits of agreement without AI were ± 8.2% and there was no interobserver variation for the AI group for the reason stated in the statistical analysis section.

#### Examination duration

Examination duration (including acquisition and measurement) was shorter in the AI group compared to the non-AI group: a median of 9.4 min (IQR 7.2–11.7) compared to 19.6 min (IQR 16.9–21.7) (*p* < 0.001).

## Discussion

The study presents, for the first time, a prospective study to evaluate the impact of an AI tool for real-time RFCSA estimation compared to the traditional manual measurement technique for monitoring muscle mass in the ICU. The AI tool succeeded in supporting operators in assessing muscle wasting in patients with a range of RFCSAs and varying image qualities by correctly delineating the RF muscle and measuring the RFCSA with less variability than standard non-AI measurements. Furthermore, the time spent on measuring RFCSA using the AI tool was approximately half that of standard measurements.

In this study, we employed a between subjects design to assess the efficacy of AI-assisted measurements in muscle ultrasound imaging against traditional manual techniques. This was necessary due to ethical and practical considerations in dealing with critically ill patients.

In our study, the observed ‘funneling’ in the Bland–Altman plots indicates a proportionate bias, particularly notable in the higher measurement errors of smaller RFCSA. This underscores the challenges inherent in the precise delineation of smaller anatomical structures. Smaller RFCSAs, due to their reduced size, are more susceptible to minor deviations during measurement, leading to proportionally greater discrepancies compared to larger RFCSAs. Such variability necessitates consideration in clinical settings, as it highlights the importance of refining measurement techniques or using AI for practitioners to ensure accuracy and reliability in ultrasound imaging of smaller muscle masses.The study showed the reduction in scan-rescan variability, this may involve both acquisition variability (taking the probe back to the same plane every time) and intraobserver delineation variability. In addition, the scan-rescan variability, intraobserver and interobserver delineation variability are similar which indicates that the main variability of the standard technique is manual delineation. This suggest that it may be possible if using the AI tool to perform a 1-scan measurement instead of standard average of 3-scan measurement, further reducing time.

Thus, with the help of the automated AI tool, monitoring of muscle changes in ICU patients could be more practical and feasible than before. The reliability of operators with limited training in our study was already good without the AI tool but the time spent on manual measurements was twice that when using the AI tool. However, further research should be focused on the use of AI to guide accurate and reproducible probe placement.

### Limitations

It is important to emphasize that removing the measure-remeasure variability and interobserver variability completely by the use of a deterministic AI model makes repeated measurements more reproducible but does not necessarily make them more accurate. Although the performance of our AI tool is good, it is essential to further assess its robustness and potential biases, especially under conditions of poor image quality. For example, conditions like sarcopenia, obesity, and severe edema may affect the AI tool’s generalizability and accuracy in clinical scenarios involving extreme physiological variations. Future research should therefore not only focus on enhancing the AI tool's reliability across different machine manufacturers and settings but also ensure its validation in diverse patient populations. This includes specific studies aimed at monitoring muscle wasting in patients with cancer and other severe conditions, to ensure the AI tool's utility and effectiveness in a broad clinical context.

The use of a between subjects design to compare non-assisted and AI-assisted measurements has its limitations. It is possible that patient characteristics, such as muscle edema, body composition (like sarcopenia, athletic build, or obesity), and muscle quality could differ between the two groups, leading to a confounding influence on the results. Although the random assignment of patients into groups minimizes this effect, it does not eliminate it completely.

Echogenicity was not investigated in this study. When muscle echogenicity increases, determining the muscle boundaries is very challenging because muscle tissue is replaced by intramuscular fibrous and fat tissue. As a result, the contrast between the muscle boundaries and other structures decreases. Future work should develop AI based methods for assessing muscle echogenicity as also muscle echogenicity can provide useful information on both quantity and quality of the muscle.

## Conclusions

Real-time AI-assisted muscle ultrasound removes the need for manual tracing, increases reproducibility, and saves time. Our study has shown that much of the variability between measurements is related to manual delineation of the muscle and hence potentially an even faster single-scan protocol could be adopted for AI-assisted RFCSA measurement. Such a system would significantly assist routine clinical monitoring of muscle changes in ICU patients and help in assessing the effectiveness of interventions.

### Supplementary Information

Supplementary Information.

## Data Availability

The datasets used and/or analyzed during the current study are available from the corresponding author on reasonable request. The codes and model weights needed to deploy the RAIMUS tool for the study are available at https://github.com/vital-ultrasound/public-muscle.
