# Supplementary material for: Clinical evaluation of AI-assisted muscle ultrasound for monitoring muscle wasting in ICU patients
Source: Sci Rep. 2024 Jun 26;14:14798. doi: 10.1038/s41598-024-64564-w (PMC11208490; doi:10.1038/s41598-024-64564-w)
Supplement: Supplementary file 1 — Supplementary Information. [file 41598_2024_64564_MOESM1_ESM.docx]

**Supplementary Appendix**

**This appendix is supplement to:** **Clinical evaluation of AI-assisted muscle ultrasound for monitoring muscle wasting in ICU patients.**

## **AI-assisted RF segmentation system**

### **Dataset**

The dataset used to train the model included 600 ultrasound images from 112 patients diagnosed with severe tetanus and central nervous infection at HTD. We randomly split the dataset into 80% for training, 10% for validation and 10% for testing. The muscle scans and manual annotations were done by three radiologists' contours of the RF performed using the built-in tracing feature of the GE Venue Go, GE Vivid IQ (GE) and handheld Phillips Lumify ultrasound machine. The characteristics of patients are shown in Table S1.

**Table S1. Characteristics of patients in the training dataset (N = 112)**

|  | **N = 112** |
| --- | --- |
| **Age** | 56 (42, 64) |
| **Sex (female)** | 25 (22%) |
| **Diagnosis** |  |
| **Tetanus** | 78 (70%) |
| **Central Nervous System Infection** | 34 (30%) |
| **Comorbidities (1 or more)** | 67 (75%) |
| **Sedative use during ICU** | 112 (100%) |
| **Use of non-depoplarising neuromuscular blocking agents during ICU stay** | 112 (100%) |
| **Length of ICU stay (days)** | 24 (17, 33) |
| **Length of hospital stay (days)** | 30 (23, 42) |
| **Mechanical ventilation duration (days)** | 18 (7, 26) |
| **Enteral nutrition** | 112 (100%) |
| **RF CSA D1 (cm^2^)** | 4.70 (3.16, 6.32) |
| **RF CSA D7 (cm^2^)** | 4.45 (3.05, 6.07) |
| **RFCSA Discharge (cm^2^)** | 3.67 (2.48, 4.97) |
| **% change in RFCSA during ICU stay (%)** | 17 (6, 32) |
| **Outcomes** |  |
| **Home** | 91 (81%) |
| **Hospital Transfer** | 100 (8.9%) |
| **Transfer To Die** | 11 (9.8%) |

### **Model architecture**

We deployed a U-net architecture [1] for the RFCSA semantic segmentation task as it has been shown to perform well for image segmentation in several medical imaging modalities even if the datasets were small. The main architecture consists of a specific contracting and an expanding path. The contracting path (or so-called decoder) consists of convolution and max pooling layers whereas the expanding path (or decoder) uses transposed convolutional layers. Skip connection was employed between layers of the same resolution in those paths. The model outputs a pixelwise binary label (background and CSA). The input images for the model were resized to 256 x 256 pixel. Augmentation was applied on-the-fly during training including rotation, horizontal flipping, zoom in/out and increase/decrease contrast gain.


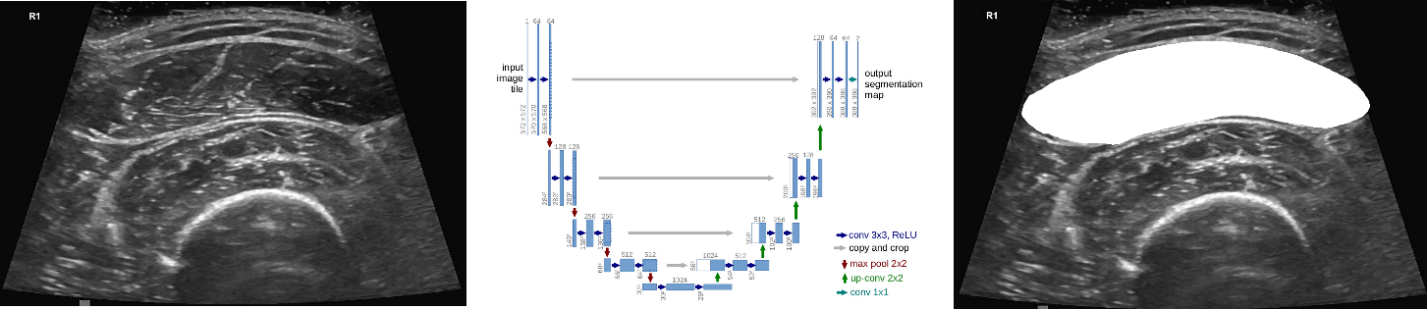


Figure S1. UNET model architecture for RFCSA segmentation

### **Training**

The model was implemented in Python 3 using PyTorch backend. It was trained using the Adam optimizer with a learning rate of 0.001. A batch size of 16 and batch normalization were applied for both CNN and transposed CNN layers. Muscle ultrasound data were augmented by adding horizontally-flipped, random rotation of 10 degrees to the training set. The models were trained on 100 epochs and evaluated on the independent test set.

### **Image pre- and post-processing**

In pre-processing, input images were resized to 128 x 128 pixels, by first resampling to isotropic pixel size (using the smallest pixel spacing as reference), followed by a padding operation to make the image square, a centered crop operation to keep the ultrasound sector in the middle and finally a resize to 128 x 128. These operations were undone in post-processing on the output segmentation mask to be able to overlay the muscle shape onto the original B-mode image.

After the pre-processing described above, input images were normalized to the range [0, 1] by dividing by the maximum feasible intensity value, 255. Output masks are in the range [0, 1] so they were scaled up to [0, 255] after postprocessing.

**Reproducibility results**

The intraobserver and interobserver ICCs are provided in Table S2, demonstrating lower intraobserver reproducibility for the manual group compared with the AI group.

**Table S2. Scan-rescan, intraobserver and interobserver variability in RFCSA measurement with and without AI**

|  | **Mode** | **ICC (95% CI)** | **SEM (95% CI), cm^2^** |
| --- | --- | --- | --- |
| **Scan-rescan variability without AI** | ACQ 1 vs ACQ 2 | 0.961 (0.936 - 0.977) | 0.30 (0.20 - 0.38) |
|  | ACQ 1 vs ACQ 3 | 0.959 (0.932 - 0.975) | 0.31 (0.22 - 0.40) |
|  | ACQ 2 vs ACQ 3 | 0.980 (0.966 - 0.988) | 0.22 (0.14 - 0.30) |
| **Scan-rescan variability with AI** | ACQ AI1 vs ACQ AI2 | 0.999 (0.999 - 0.999) | 0.04 (0.02 - 0.05) |
|  | ACQ AI1 vs ACQ AI3 | 0.999 (0.998 - 0.999) | 0.04 (0.03 - 0.04) |
|  | ACQ AI2 vs ACQ AI3 | 0.999 (0.998 - 0.999) | 0.04 (0.03 - 0.04) |
| **Intraobserver variability without AI** | DRs measure vs DRs remeasure | 0.984 (0.973 - 0.990) | 0.19 (0.14 - 0.24) |
| **Interobserver variability without AI** | DR1 vs DR2 | 0.974 (0.965 - 0.981) | 0.25 (0.21 - 0.28) |
|  | DR1 vs DR3 | 0.972 (0.962 - 0.979) | 0.26 (0.22 - 0.30) |
|  | DR2 vs DR3 | 0.978 (0.971 - 0.984) | 0.23 (0.20 - 0.26) |

*(ICC: Intraclass correlation, MD: Mean Difference, SEM: Standard Error of Measurement, ACQ AI: Acquisition using AI tool)*
